# The practices of nurses about palliative sedation on palliative care wards: A qualitative study

**DOI:** 10.1111/jan.15350

**Published:** 2022-06-30

**Authors:** Linda Heino, Minna Stolt, Elina Haavisto

**Affiliations:** ^1^ Department of Nursing Science University of Turku Turku Finland; ^2^ Department of Nursing Science University of Turku, Turku University Hospital Turku Finland; ^3^ Department of Health Sciences Tampere University, Tampere University Hospital Tampere Finland; ^4^ Satakunta Hospital District Pori Finland

**Keywords:** hospice care, nurses, nursing, palliative care, sedation

## Abstract

**Aim:**

To describe the practices of nurses concerning palliative sedation in palliative care wards in hospitals.

**Design:**

Qualitative descriptive design.

**Methods:**

A total of 27 nurses were interviewed in six focus groups and one pair interview; the nurses worked on four palliative care wards in three Finnish hospitals and the interviews took place between May and November 2019. The data were analysed using inductive content analysis. The COREQ checklist was used as a guide for reporting this study.

**Findings:**

Three main categories describing nurses' practices concerning palliative sedation were identified: participation in palliative sedation decision‐making, sedation implementation and monitoring and information sharing and compassionate care for the patient and relatives.

**Conclusions:**

Nurses play a key role throughout palliative sedation on palliative care wards in hospitals. To develop the quality of care, it is recommended to specify the role of nurses in palliative sedation, increase cooperation between nurses and physicians, and enhance palliative sedation education for nurses.

## 1 INTRODUCTION

Palliative sedation is used in palliative care as a last resort to alleviate intolerable and otherwise uncontrollable physical (i.e., pain) and non‐physical (i.e., psychological, emotional) suffering (Cherny & Radbruch, 2009). Despite advances in palliative care, some dying patients still suffer from unbearable symptoms that are not alleviated by conventional treatment options (Hui et al., 2015). The most frequent indications for palliative sedation are delirium, pain and dyspnoea (Arantzamendi et al., 2021).

There is no precise information on the prevalence of the need for palliative sedation. However, according to one literature review, around 25%–33% of all terminally ill patients undergo palliative sedation, and approximately 25%–33% of them need continuous deep sedation (Garetto et al., 2018). The European Association for Palliative Care (EAPC) has issued a recommendation on the use of palliative sedation (Cherny & Radbruch, 2009), in addition to which several other guidelines are also available (Abrashi et al., 2017). Even though the use of palliative sedation appears to be quite common and recommendations for its use exist, palliative sedation and in particular the difference between palliative sedation and euthanasia remains a much‐discussed topic in the context of palliative care (Riisfeldt, 2019). In addition, there is a lack of consensus about the use of palliative sedation in the treatment of psychological or existential distress (Cherny & Radbruch, 2009). Furthermore, there is still variation between expert recommendations (Abrashi et al., 2017) and the implementation of palliative sedation in practice (Seymour et al., 2015).

Nurses' role in palliative sedation is crucial (Heino et al., 2021). However, studies about the practices of nurses related to palliative sedation are rare. To develop the quality of care, it is essential to understand the practices of nurses about palliative sedation. As there is little prior knowledge on the topic, it is relevant to conduct the research by using an exploratory and descriptive qualitative approach. The findings of qualitative research can be employed in the planning, implementation and management of palliative sedation in units where palliative care patients are treated, as well as when making recommendations for education and guidelines concerning palliative sedation (Leeman & Sandelowski, 2012).

### 1.1 Background

The EAPC has defined palliative sedation as “the monitored use of medications intended to induce a state of decreased or absent awareness (unconsciousness) in order to relieve the burden of otherwise intractable suffering in a manner that is ethically acceptable to the patient, family and health‐care providers” (Cherny & Radbruch, 2009). This definition is used in the context of this study, although a universal definition for the concept of palliative sedation is still lacking. In this definition, different types of palliative sedation can be distinguished, i.e., intermittent and continuous as well as superficial and deep sedation, and the definition also covers emergency sedation (Cherny & Radbruch, 2009). The different types of palliative sedation are described in Table 1. According to the guidelines of EAPC, continuous deep sedation should only be considered in situations where the expected prognosis of the patient is hours or days at the most (Cherny & Radbruch, 2009).

**TABLE 1:** Different types of palliative sedation based on the EAPC recommended framework for the use of sedation in palliative care (Cherny & Radbruch, 2009)

The type of sedation	Definition
Continuity	
Intermittent	Sedation is performed for a limited time to provide temporary relief
Continuous	Medicines are given continuously to achieve a persistent effect
Depth	
Superficial	The patient retains, at least in part, the ability to communicate with family or caregivers
Deep	The patient is completely unconscious
Emergency sedation	Sedation in emergencies such as sudden massive haemorrhage or shortness of breath

The findings of a previous scoping review showed that nurses play key roles in providing palliative sedation as they often participate in decisions concerning the start of sedation, administer the medication and monitor sedation, as well as provide information and compassionate care to the patient and relatives (Heino et al., 2021). Because nurses are usually the health care providers who spend the most time in the presence of the patient, they are in a unique position to assess the need for palliative sedation (Anquinet et al., 2015; Zinn & Moriarty, 2012; Zuleta‐Benjumea et al., 2018). However, sedation guidelines do not usually define the precise role of nurses in palliative sedation decision‐making (e.g., Cherny & Radbruch, 2009), and it has been recognized that the involvement of nurses in the decision to initiate palliative sedation varies significantly between countries (Anquinet et al., 2015), care settings (Arevalo et al., 2013; Inghelbrecht et al., 2011) and cases (Inghelbrecht et al., 2011).

It has been reported that, at least in the Netherlands, Belgium and the UK, nurses often initiate palliative sedation without the presence of a physician (Anquinet et al., 2015; Arevalo et al., 2013; Brinkkemper et al., 2011), although the EAPC recommends that palliative sedation should be commenced in cooperation with the physician and the nurse whenever possible (Cherny & Radbruch, 2009). In addition, monitoring the effectiveness of the sedation also seems to be primarily the nurse's task (e.g., Anquinet et al., 2015; Brinkkemper et al., 2011; Zuleta‐Benjumea et al., 2018). However, studies describing how nurses monitor sedation are scarce. In addition, there is no evidence suggesting that nurses base the monitoring on any guidelines or use any specific clinical evaluation tools during the monitoring. Nevertheless, it has been recognized that, in some cases, nurses make independent decisions about the patient's medication and dosage policy based on monitoring (Brinkkemper et al., 2011; De Vries & Plaskota, 2017; Zinn & Moriarty, 2012). Finally, there is little research on nurses' practices in relation to emergency sedation or fundamental care of the patient during palliative sedation.

The literature shows that nurses, together with other team members, are involved in informing both the patient and relatives about palliative sedation (e.g., Dwyer & McCarthy, 2016; Zinn & Moriarty, 2012; Zuleta‐Benjumea et al., 2018). Nurses believe that the patient and relatives need to be given this information several times to facilitate understanding (Zuleta‐Benjumea et al., 2018). According to nurses, it is important to be aware of the necessity to explain the reality of the situation (Zinn & Moriarty, 2012), and the need to do this in a sensitive manner (Dwyer & McCarthy, 2016). The provision of information to the patient and relatives is also emphasized in the recommendations on palliative sedation (e.g., Cherny & Radbruch, 2009). However, little is known about what information nurses provide to the patients and relatives before sedation and to relatives during sedation. In addition to providing information, nurses also provide the patient and family with compassionate care (Venke Gran & Miller, 2008). It has been shown that especially relatives may experience significant anxiety due to the initiation of palliative sedation (Bruinsma et al., 2012), and the guidelines about palliative sedation also stress the importance of supporting relatives (e.g., Cherny & Radbruch, 2009). However, little is known about how nurses provide compassionate care to the patient and relatives in the context of palliative sedation.

More research on the practices of nurses about palliative sedation is required. Research is still scarce, and some practices of nurses appear to be contrary to the recommendations, as well as considerable differences existing between countries, care settings and cases. In addition, it is important to learn more about the practices of nurses because some nurses report an emotional burden due to ethical uncertainties associated with administering palliative sedation (e.g., Anquinet et al., 2015; De Vries & Plaskota, 2017; Dwyer & McCarthy, 2016). Therefore, this study aims to explore this gap in the research about the practices of nurses related to palliative sedation.

## 2 THE STUDY

### 2.1 Aim

The aim of this study was to describe the practices of nurses concerning palliative sedation in palliative care wards in hospitals. The main goal was to deepen the understanding of the topic to improve the quality of care. The research question was as follows:

1. What practices are included in the role of nurses in palliative sedation?

### 2.2 Design

This study was conducted using a qualitative descriptive study design to obtain rich descriptions of the practices of nurses concerning palliative sedation from the perspective of the nurses implementing the treatment (Bradshaw et al., 2017).

### 2.3 Sample/participants

In the Finnish model, palliative care and hospice care are divided into a basic level (all social and health care units treating dying patients and not belonging to ABC‐levels), an A‐level (hospice care units at the basic level), a B‐level (special level palliative care and hospice care units) and a C‐level (the palliative centres of university hospitals providing demanding special level palliative care). Most patients are treated at the basic level or A‐level, but about a third of patients require level B or C treatment. Palliative sedation is mainly used in special care units. Furthermore, the profession of nurse is a profession that is legalized in Finland. This profession may only be practised by a registered professional, therefore, only Registered Nurses work in palliative care and perform palliative sedation.

The data were collected in specialized care (B‐level) units in Finland from three hospitals and four different wards. The hospitals were located in an area that covers two hospital districts comprising about 40% of the Finnish population. A purposive sampling method was used to find nurses with experience of palliative sedation (Holloway & Wheeler, 2010). Nurses met the inclusion criteria if they were Finnish‐speaking and Registered Nurses working on wards where palliative sedation is performed. Prior to the actual recruitment, the author and/or interviewer visited the target organizations to personally inform potential participants about the study, in addition to which written material about the study was left on the wards. After being informed about the study, the liaison officers on the wards recruited participants. Once the participants had been recruited, the researchers agreed on an appropriate time for interviews with the liaison officers on the wards.

The number of focus groups was not predetermined, but 4–8 participants were targeted for one group. All the nurses in the four wards met the inclusion criteria, and they were all invited to participate in the study to obtain the most comprehensive description of the topic as possible. Of the total of 50 Registered Nurses who were invited, 27 participated (54%). Nurses who worked in the same ward were in the same focus group. Table 2 presents an overview of the characteristics of the participants.

**TABLE 2:** Characteristics of the participants (*n* = 27)

Variables	N	Mean	Median	SD	Range	f (%)
Age (years)	27	42.2	45.0	11.0	24–60	
In‐service training in palliative care	27					
Yes						19 (70.4)
No						8 (29.6)
Work experience in health care (years)	27	15.8	12.6	11.6	0.8–37	
Work experience in palliative care (years)	26	5.3	3.8	5.0	0.3–19	
Experience with palliative sedation in the last 6 months (cases)	25	4.0	3.0	4.6	0–20	

Abbreviation: SD, Standard Deviation.

### 2.4 Data collection

The data were gathered using semi‐structured focus group interview methods as there was a desire to stimulate ideas and elicit feelings about the topic of the study through dynamic interaction as well as to observe agreement and differences of opinions amongst participants (Holloway & Wheeler, 2010). A total of six focus group interviews (three to six participants) and one pair interview (other recruited nurses were unable to attend) were conducted between May and November 2019 (Table 3). The duration of the interviews ranged from 45 to 173 min (mean 75 min). The interview guide consisted of seven themes that had emerged from key findings in the prior literature (Heino et al., 2021). The themes concerned (1) decision‐making about palliative sedation, (2) compassionate care for the patient, (3) administration of medication and monitoring the sedation, (4) fundamental care of the patient, (5) compassionate care for the family, (6) providing information for the family during sedation and (7) cooperation between nurses and physicians throughout sedation. The interview questions were formed on the basis of these themes (Table 4).

**TABLE 3:** Description of data collection

Interview type	Number of participants
Focus group FG1	6
Focus group FG2	5
Focus group FG3	4
Focus group FG4	4
Focus group FG5	3
Focus group FG6	3
Pair interview PI1	2

**TABLE 4:** The interview guide

Theme	Questions
The decision‐making about palliative sedation	How is the decision to start sedation made? (In what situation?) What is the role of the nurse in the decision‐making? What kind of discussions take place with the patient and loved ones before palliative sedation? How is information shared? How are decisions made during sedation? How are the decisions on changes in sedation made? (e.g., dose levels) How is the consultation done? (e.g., physician, another nurse)
Compassionate care for the patient	How is the patient supported before sedation? How are the needs and wishes of the patient considered?
Administration of medication and monitoring the sedation	How is the sedation started? How does the situation progress after starting sedation? How is sedation monitored?
Fundamental care of the patient	How is the patient's fundamental care performed during sedation?
Compassionate care for the family	How are relatives supported before sedation? How are relatives supported during sedation? How are the needs and wishes of relatives considered?
Providing information for the family during sedation	How is information shared with relatives and how are relatives guided during sedation?
Cooperation between nurses and physicians throughout sedation	What kind of cooperation is there between the physician and the nurse in palliative sedation?

The data were collected by two female interviewers, one of whom was a PhD student and the other a nurse with a Bachelor of Nursing Science degree (BNSc). Both interviewers had experience in conducting a research interview; in addition, the BNSc interviewer worked as a nurse in palliative care and had considerable experience with palliative sedation. Both interviewers were unfamiliar with the interviewees. However, the role and position of the interviewer in the research project was communicated to the participants before the interviews. The focus groups/interviews were conducted by one interviewer in private spaces in the workplace of the participants so that only the interviewees and the interviewer were present. All data were audio‐taped with the permission of the participants to ensure precise records of the participants' accounts. No repeat interviews were conducted.

### 2.5 Ethical considerations

The research adhered to the ethical principles of the Declaration of Helsinki (World Medical Association, 2013). An Ethics committee approval was obtained from a university (15/2019), and research permits were granted by each target organization. Participation in the study was voluntary, and it was possible to suspend participation at any time without providing a reason. The organizations were responsible for recruiting suitable interviewees, and the researchers were not able to influence the selection. Participants were informed about the study orally and in writing. Each interviewee signed informed consent to participate in the study. The privacy of the participants and the confidentiality of their personal information were ensured throughout the study, and individuals or organizations cannot be identified from the quotations used.

### 2.6 Data analysis

The data were analysed with inductive content analysis as is recommended when there is little previous knowledge about the phenomenon (Elo & Kyngäs, 2008). After transcription of the data, the material was read through several times to obtain an overall view. Furthermore, the data were searched for units of analysis, i.e., sentences related to the research question. These units of analysis were then abridged into simpler expressions to facilitate analysis. The simplified expressions were coded, and similar codes were classified into subcategories. Similar subcategories were then combined into categories, and categories were grouped into main categories (Table 5). Data analysis was conducted without any software by one author (BNSc, RN with expertise in palliative care) under the mentorship of the other authors (PhD, Docent and PhD, Professor), all of whom are women.

**TABLE 5:** An example of the formation of the main categories

Original expression	Simplification	Subcategory	Category	Main category
The medication instructions come from the physician, the nurse prepares the pump.	The nurse prepares the pump according to the physician's instructions.	Preparing and mounting the sedation pump	Initiating sedation	Sedation implementation and monitoring
Maybe a single dose of the drug is given initially, and at the same time, a pump with more of the same medication is attached according to the physician's instructions, i.e. the infusion rate.	A single dose of the medicine may be given and at the same time a pump attached with the infusion rate set according to the physician's instructions.			
Crisis medications are likely to be repeated twice and then perhaps the original pain pump proves sufficient.	Crisis medications are usually given twice.	Administration of emergency drugs		

### 2.7 Rigour

The processes described by Elo et al. (2014) were applied in the preparation, organization, and reporting phases to establish the trustworthiness of the study. A purposive sampling method was used as there was a desire to interview people who had the best knowledge about the research topic. A focus group interview method was an appropriate method for gathering data because the group processes could help the researcher understand the reality described by the participants. The participants were recruited from four different palliative care wards to achieve various perspectives. To facilitate the evaluation of the analysis, the analysis unit simplifications and abstractions have been illustrated and presented in Table 5. The data were analysed by only one researcher, but the other authors monitored the analysis process, and the subcategories, categories and main categories were discussed in the research group and consensus was achieved. Transferability was eased by providing descriptions of the context of the study as well as the selection and characteristics of participants. Furthermore, appropriate quotations translated into English by the author were used to indicate the trustworthiness of the findings. To enable readers to fully appraise the study, we followed the Consolidated Criteria for Reporting Qualitative Research (COREQ) checklist for qualitative studies (Tong et al., 2007).

## 3 FINDINGS

The characteristics of the 27 participants are presented in Table 2. The age of the participants varied from 24 to 60 years. Nineteen nurses (70%) had in‐service training in palliative care. The participants' work experience in health care varied from 10 months to 37 years and in palliative care, from 3 months to 19 years. Regrettably, one participant's work experience in palliative care was not known. The participants' experience of palliative sedation in the last 6 months ranged from zero to 20 cases. Two of the interviewees did not give an exact number, but they described having performed palliative sedation several times in the last 6 months.

Three main categories describing nurses' practices about palliative sedation were identified: (1) participation in palliative sedation decision‐making, (2) sedation implementation and monitoring, and (3) information sharing and compassionate care for the patient and relatives (Table 6).

**TABLE 6:** The practices of nurses about palliative sedation

Main categories	Categories	Subcategories
Participation in palliative sedation decision‐making	The conditions for applying palliative sedation as experienced by the nurses Shared decision‐making The ethical issues experienced by the nurses participating in the decision‐making	Intolerable suffering Last resort option Limited life expectancy The desire of the patient Raising the question of sedation with the patient and the family Identifying the need for sedation and reporting it to the physician Deciding on the use of a prescribed prescription Challenges related to sedation indications Patient's inability to participate in decision‐making
Sedation implementation and monitoring	Initiating sedation Monitoring during sedation Administration of additional drugs and implementation of medication changes Fundamental care of the patient during sedation	Preparing and mounting the sedation pump Administration of emergency drugs Comprehensive monitoring Reporting the findings of monitoring to the physician Administration of additional drugs Changing medication Fundamental care is delivered in a similar manner as when awake Fundamental care is delivered lightly and calmly
Information sharing and compassionate care for the patient and relatives	Information sharing and compassionate care for the patient and relatives before sedation Information sharing and compassionate care for relatives during sedation	Sharing information with the patient and relatives before sedation Providing compassionate care for the patient and relatives before sedation Sharing information with relatives during sedation Providing compassionate care for relatives during sedation

### 3.1 Participation in palliative sedation decision‐making

*Participation in palliative sedation decision‐making* includes the conditions for applying palliative sedation as experienced by the nurses, shared decision‐making, and the ethical issues experienced by the nurses participating in the decision‐making (Table 6).

#### 3.1.1 The conditions for applying palliative sedation as experienced by the nurses

*Intolerable suffering* refers to severe pain, dyspnoea, nausea, anxiety, or existential distress, which nurses reported as typical reasons for palliative sedation. In addition, according to the nurses, emergency situations can include massive bleeding, severe shortness of breath or severe pain.

In severe pain, severe dyspnoea, bleeding if there is massive bleeding. (FG5).

*Last resort option* means that according to the nurses' experience, palliative sedation is used only when conventional treatments do not relieve the intolerable symptoms.

All other medicines have by then been used. (FG4).

*Limited life expectancy* means that the initiation of continuous sedation, based on the nurses' experience, requires that death is expected to occur in the very near future.

After all, we cannot sedate a patient until death if he or she is not yet close to death. (FG1).

*The desire of the patient* suggests that, according to the nurses, it is important to ensure that the patient himself/herself wants to be sedated before initiating palliative sedation.

I have to say that the most important thing is… first you have to make sure that the patient really wants it. (FG1).

#### 3.1.2 Shared decision‐making

*Raising the question of sedation with the patient and the family* suggests that if the patient does not broach palliative sedation himself/herself, it is usually either the physician or the nurse who starts the discussion about sedation with the patient and the family. Sedation is considered especially when the patient has a strong fear or when massive bleeding or suffocation is expected.

Yes, we may then tell the patient if it is known that somehow uncomfortable symptoms are to be expected, or… lung cancer patients, at least I have told everyone that there is such a possibility. (FG1).

*Identifying the need for sedation and reporting it to the physician* means that the practices of nurses in the decision‐making include identifying the patient's need for sedation, possibly discussing it with relatives and referring this information to the physician. Nurses spend considerable time with the patient, which is why they often see the patient's intolerable suffering before the physician. In some cases, usually after discussion with colleagues, the nurse may propose sedation to the physician, who has the ultimate responsibility for the sedation decision.

Since we are with these patients 24/7, we are pretty much aware of the situation. (FG2).

It is such an important decision that I usually talk to my colleagues before recommending it to the physician. (FG2).

*Deciding on the use of a prescribed prescription* suggests that the nurse together with colleagues may decide to introduce emergency drugs or to start the sedation pump in cases where the physician has written a prescription in advance.

If, for example, at the weekend when we do not have our own physician here, there is often a situation where we are wondering whether we should introduce the emergency drugs or not, in which case we make the decisions together… (FG6).

#### 3.1.3 The ethical issues experienced by the nurses participating in the decision‐making

*Challenges related to sedation indications* suggest challenges related to the definition of intolerable suffering and the timing of sedation. The patient, physician and nurse do not always agree on the onset of palliative sedation.

Who can define what is intolerable suffering? (FG1).

I have felt like the physician is afraid to make the decision, and I have seen situations where sedation was not implemented but might have been appropriate. (FG6).

*Patient's inability to participate in decision‐making* refers to challenges related to the inability of the patient to participate in the palliative sedation decision due to confusion or decreased level of consciousness.

Of course, if the patient is very delirious or so… in that situation, he or she may not be able to make or express a conscious decision about what his/her own wish is at that point. (PI1).

### 3.2 Sedation implementation and monitoring

*Sedation implementation and monitoring* includes initiating sedation, monitoring during sedation, administration of additional drugs and implementation of medication changes and fundamental care of the patient during sedation (Table 6).

#### 3.2.1 Initiating sedation

*Preparing and mounting the sedation pump* refers to the fact that initiating the pre‐planned palliative sedation in practice is generally performed independently by the nurse. The medication, prescribed by the physician, is individual, but the typical sedating drugs used are midazolam and dexmedetomidine. In addition, the patient usually receives a painkiller and, if necessary, other medications. The drugs are usually administered subcutaneously, but other routes of administration, such as intravenous, may be used if necessary. When attaching a sedation pump, the nurse typically administers a single dose of the sedating drug to accelerate the onset of the sedation effect. If possible, continuous sedation is started in the daytime when a physician is present on the ward.

Maybe a single dose of the drug is given initially, and at the same time, a pump with more of the same medication is attached according to the physician's instructions, i.e., the infusion rate. (PI1).

*Administration of emergency drugs* refers to the fact that giving emergency medication in a catastrophic event is generally performed independently by the nurse. Emergency drugs are given as prescribed by the physician, usually as repeated injections. Nurses are often aware of the opportunity of an emergency in advance and are prepared for it.

We have a certain procedure: a certain drug is given every 15 or 10 min until the situation gets better, we have good instructions from the physician. And we actually know in advance, the physician has already told us that this kind of thing has been diagnosed in this disease, i.e., there may be suffocation death here… (FG2).

#### 3.2.2 Monitoring during sedation

*Comprehensive monitoring* means that the practices of nurses during palliative sedation include the frequent and holistic monitoring of the patient, with a particular focus on the depth of sedation and pain. Monitoring is particularly frequent at the beginning of sedation. Things to monitor during sedation include breathing, skin colour, vocalization, expressions, stiffness, restlessness and responding to speech or touch. The use of the Pain Assessment in Advanced Dementia Scale (PAINAD) was mentioned for pain monitoring. In addition to nurses, the physician and relatives also participate in the monitoring.

We monitor and see if he or she wakes up to speech and touch, and we make sure he or she does not look as if in pain. (FG3).

*Reporting the findings of monitoring to the physician* suggests that the nurse should actively tell the physician what was observed during the monitoring. A possible on‐call physician will not always have adequate knowledge about palliative sedation, so it is important for the nurse to be able to propose the necessary medication changes.

Whether sedation is adequate, whether pain management is adequate, or what the patient's situation is, we take that information to the physician. (FG3).

#### 3.2.3 Administration of additional drugs and implementation of medication changes

*Administration of additional drugs* means that the practices of nurses during sedation include deciding on the need for additional drugs and administering the necessary additional drugs in accordance with the instructions, usually prescribed in advance by the physician. The additional drugs can be given by different routes of administration, often as boluses from a sedation pump or as separate injections. Nurses feel that deciding on additional sedative drugs is particularly challenging in situations where there is no clear decision on and goal for palliative sedation.

Boluses and short‐acting drugs are always included when the treatment is implemented. (FG1).

Because there have now been many cases where no decision has been made on palliative sedation; however, the Dexdor pump is started at a fairly high dose to keep the patient calm and asleep, but there is no decision that the patient should stay asleep… Then we wonder whether that patient can be awake, or whether we should start medicating him or her right now. (FG2).

*Changing medication* means that the practices of nurses during sedation include making the necessary medication changes to achieve and maintain the desired level of sedation and keep the patient asymptomatic. The physician often prescribes instructions for changing the medication in advance, and the nurse decides when the changes will be implemented and implement them accordingly. Finding the right medication and dosage is often challenging, requiring constant dialogue between the physician, nurses and relatives.

However, sedation medications are usually started at a slightly lower dose… and if there is no response, the dose is increased. (FG2).

We monitor the patient's condition, and the physician gives the instructions to reduce or increase the amount of medication. (PI1).

#### 3.2.4 Fundamental care of the patient during sedation

*Fundamental care is delivered in a similar manner as when awake* means that the patient's fundamental care performed by the nurse changes little after the initiation of sedation. The fundamental patient care during sedation includes, for example, position changes, hygiene, oral care and eye care. However, fundamental care is always individualized depending on the patient's condition. During fundamental care, the patient is treated with respect; the patient is stroked, and he or she is still talked to and told what is being done.

We make position changes and all that… when the patient cannot change position for himself/herself, it's good to have regular position changes and use air mattresses and other things like that. (FG3).

*Fundamental care is delivered lightly and calmly* suggests that the nurse carries out the patient's fundamental care in a way that does not disturb the patient's sleep or increase symptoms. Extensive washing, bowel emptying and so on are avoided during sedation. Prior to sedation, the patient is usually given a permanent catheter to avoid urinary retention and major position changes.

Very lightly, maybe just those necessary. (FG6).

### 3.3 Information sharing and compassionate care for the patient and relatives

*Information sharing and compassionate care for the patient and relatives* includes information sharing and compassionate care for the patient and relatives before sedation, and information sharing and compassionate care for relatives during sedation (Table 6).

#### 3.3.1 Information sharing and compassionate care for the patient and relatives before sedation

*Sharing information with the patient and relatives before sedation* refers to the provision of information by the nurse to the patient and the family prior to palliative sedation. The information related to palliative sedation is provided several times by both the physician and nurses before the sedation is started. The information to be conveyed to the patient and relatives before sedation includes the goals, initiation and course of sedation and the operation of the sedation pump. Other issues include the monitoring of pain during sedation, the differences between sedation and anaesthesia and the possibilities of waking the patient and the patient awaking during sedation. In the case of continuous deep sedation, relatives often fear that the sedation will promote death as the patient is no longer able to drink. It is, therefore, important to tell them that the process of death is already underway in any case and to also provide information about the disadvantages of hydration during sedation.

Well maybe what follows after this, or what happens now and how it happens, and for example, that the patient does not fall asleep right away, but might just fall asleep properly after several hours. And there may be occasional unintentional awakenings from time to time. And what happens after that and at the start, and how I start it and how that pump works and so on. (FG2).

*Providing compassionate care for the patient and relatives before sedation* refers to the nurses' actions to reduce the worries and anxiety of the patient and the family before sedation. The patient support before sedation is quite similar to the previous support. In the case of continuous deep sedation, the nurse seeks to fulfil the patient's wishes and encourages him/her to take care of the things he or she needs to before sedation. Furthermore, it is important for the nurse to ascertain the patient's and relatives' wishes about when to start sedation and allow relatives to say goodbye.

And if there is something he or she wants to do, or someone he or she wants to talk to, or what he or she wants to eat before the sedation. If there is no urgency in that situation, emergency sedation is a different matter, but in such a peaceful situation, attempts are made to fulfil those wishes. (FG2).

…before we begin, it is important that… all the patient's visitors can have the opportunity to say goodbye. (FG3).

#### 3.3.2 Information sharing and compassionate care for relatives during sedation

*Sharing information with relatives during sedation* refers to the continuation of information related to palliative sedation provided to the family by the nurse. The issues that relatives need to be told during sedation include, for example, that the patient's vocalization and movement are normal during sedation, and that the patient often does not suffer from certain symptoms such as mucus retention during sedation, even if the situation looks bad to other people, and that it is possible to increase the medication if required. In the case of continuous deep sedation, providing information in advance about impending death and its signs, such as intermittent breathing and a death rattle, is important to reduce relatives' fear. In addition, the nurse also informs the relatives of the significance of their presence next to the patient. Relatives are told that even if the patient may be unable to communicate, he or she may still hear and feel touch. Furthermore, the nurse encourages relatives to ask questions and answers them honestly, although sometimes no one knows the answer.

It is told that the patient does not suffer from those symptoms, although they may seem bad to us, but quite often patients do not feel it as something bad and do not feel the mucus during sedation, for example. (FG3).

We try to tell relatives that it is important that they are present despite the fact that the patient is asleep. (FG2).

*Providing compassionate care for relatives during sedation* refers to the continuation of compassionate care provided to the family by the nurse. During continuous deep sedation especially, the need for support from relatives is emphasized as relatives are usually quite frightened when the patient's death is imminent. The nurse can support relatives by being present, talking to and listening to them. In addition, the nurse can reduce the anxiety of relatives by saying things related to the patient's condition out loud and emphasizing the patient's calmness. In addition, the nurse will discuss the ability of the relatives to cope as well as reminding and encouraging them to take care of themselves by eating and sleeping regularly. Furthermore, nurses will encourage relatives to touch and stroke the patient, talk to him/her and participate in the treatment activities, such as wetting the patient's mouth.

And then… the shoulder can always be stroked, and you can ask: “Is it bad for you to be here, is it scary for you to be here next to the patient?” You can ask that. “I can stay here with you for a while. There is nothing to fear here.” You can then stroke the patient. “He or she is sleeping, he or she is looking calm, our medication has been successful, now he or she does not have to suffer at all.” (FG1).

## 4 DISCUSSION

The aim of this study was to describe the practices of nurses concerning palliative sedation in palliative care wards in hospitals. Earlier studies have shown that nurses play a key role in palliative sedation, but the research is still limited. The main categories identified in this study that describe nurses' practices about palliative sedation were: participation in palliative sedation decision‐making; sedation implementation and monitoring; information sharing and compassionate care for the patient and relatives. This study confirmed the findings of the previous review (Heino et al., 2021), and added evidence, particularly of nurses' roles in sedation monitoring, emergency sedation, fundamental care of the patient during sedation and providing information and compassionate care to patients and relatives. The findings of this study will increase understanding of the practices of nurses concerning palliative sedation, which may contribute to the improvement of the quality of care.

In this study, nurses described that the sedation decision was usually made in a collaboration between the patient, relatives, physician, and nurses, although the final responsibility is that of the physician. It became obvious in the interviews that nurses had an important role, especially in identifying the need for sedation and reporting the symptoms to the physician. This finding corresponds with the findings of earlier research, according to which determining the need for sedation is a key task of the nurse in decision‐making (Anquinet et al., 2015; Zinn & Moriarty, 2012; Zuleta‐Benjumea et al., 2018). However, the Finnish nurses in this study were cautious about proposing sedation to the physician, in contrast to British nurses (Anquinet et al., 2015). In addition, this study revealed that in some cases the practices of nurses in the decision‐making included raising the question of sedation with the patient and relatives. However, in the previous study, it was the physician who most often discussed the option of palliative sedation with the patient and the family (Anquinet et al., 2015). Moreover, it was noticed in this study, as well as in a previous one (Anquinet et al., 2015), that nurses could decide independently when to start sedation if the physician had written a prescription in advance. However, in the present study, nurses were cautious about making the decision to start sedation and emphasized the importance of collegial support. Overall, the cautious attitude of Finnish nurses in palliative sedation decision‐making is probably due to ethical and legal challenges relating to palliative sedation, especially the fact that, if misused, sedation may hasten the patient's death. In addition, the EAPC recommendation, which guides the use of sedation in palliative care in Finland, does not particularly focus on the practices of nurses in palliative sedation decision‐making (Cherny & Radbruch, 2009).

The crucial role of nurses in sedation implementation and monitoring was highlighted in this study. The nurses described that they generally started the syringe driver in the absence of the physician, in agreement with previous studies (Anquinet et al., 2015; Arevalo et al., 2013; Brinkkemper et al., 2011). In addition, in this study, the nurses also described administering emergency drugs, usually independently. However, the independent initiation of sedation by a nurse is contrary to the EAPC recommendations (Cherny & Radbruch, 2009). About sedation monitoring, consistent findings concerning the major role of nurses in assessing the effectiveness of the sedation were presented in previous studies (e.g., Anquinet et al., 2015; Brinkkemper et al., 2011; Zuleta‐Benjumea et al., 2018). However, there was little previous knowledge of how nurses conducted the monitoring. This study produced new insights related to sedation monitoring as nurses described monitoring as frequent and comprehensive, and described observing several things in the patient and evaluating his or her response to speech or touch. However, the monitoring described by nurses in this study was inconsistent: the exact frequency was not mentioned at all, nor were any follow‐up measures, except for the PAINAD measure in pain monitoring. It was quite surprising that although patient monitoring after initiation of palliative sedation is guided by various clinical guidelines (e.g., Cherny & Radbruch, 2009) and there are several instruments to assess refractory symptoms and the effects of palliative sedation (Belar et al., 2021), there was no indication in this or the previous studies where monitoring was described (Brinkkemper et al., 2011; Zuleta‐Benjumea et al., 2018) that nurses perform monitoring based on these guidelines or instruments. Nevertheless, in agreement with earlier research (Brinkkemper et al., 2011; De Vries & Plaskota, 2017; Zinn & Moriarty, 2012), the nurses in this study described making independent decisions concerning additional drugs and medication changes on the basis of monitoring. Finally, this study produced unique findings as regards the fundamental care of the patient and the special features required during palliative sedation; this is a topic about which there was almost no prior knowledge, but which is also an important aspect related to palliative sedation (Cherny & Radbruch, 2009).

Sharing information and providing compassionate care for the patient and relatives were described as important practices for nurses in this current study. Consistent findings in terms of information (e.g., Dwyer & McCarthy, 2016; Zinn & Moriarty, 2012; Zuleta‐Benjumea et al., 2018) and compassionate care (Venke Gran & Miller, 2008) provided by nurses were also presented in previous studies. However, this study produced unique findings related to issues to be communicated to the patient and relatives before sedation and to issues to be communicated to relatives during sedation, about which there was little previous information. These issues concerned, for example, the course of sedation, the differences between sedation and anaesthesia, and impending death. In addition, this study confirmed the previous knowledge that information should be provided to family members several times (Zuleta‐Benjumea et al., 2018) and as truthfully and kindly as possible (Dwyer & McCarthy, 2016; Zinn & Moriarty, 2012). Furthermore, the current study introduced novel findings on the ways in which the nurse can provide compassionate care to both the patient and relatives. These included, for example, fulfilling the patient's wishes before sedation, allowing the family to say goodbye, and supporting the family during sedation by being present, listening to them and emphasizing the patient's calmness. In agreement with a previous study (Venke Gran & Miller, 2008), this study also found that nurses instructed relatives to talk to and touch the patient and to assist in treatment activities.

To enhance the quality of care, first, it is recommended that the practices of nurses in palliative sedation be clarified in clinical practice as well as in the palliative sedation guidelines. It is essential to define the role and responsibilities of nurses especially in palliative sedation decision‐making, implementation, and follow‐up. In terms of decision‐making, it is important to determine, for example, who should raise the issue of sedation with the patient and relatives and who should propose this treatment to them. This is important because professionals may have different perceptions of the need for sedation as well as what the concept of palliative sedation means. In connection with the implementation and monitoring of sedation, it is important to specifically determine, who should be present at the start of sedation and how and by whom the sedation is to be monitored.

Second, it is suggested that the cooperation between nurses and physicians is increased. The nurse's view of the need for sedation should be strongly considered and nurses should be able to suggest sedation to the physician without trepidation. In addition, a physician with expertise in palliative sedation should be involved more often in initiating sedation and available for consultation during sedation. Moreover, it is important that the physician clearly records the sedation decision and the purpose of the sedation so that the nurse can make decisions about giving additional drugs and making changes to the medication. Third, it is suggested that more palliative sedation education is offered to nurses who carry out the treatment. This training is important so that nurses have sufficient knowledge to carry out and monitor sedation and provide information and compassionate care to patients and relatives. More education is needed especially related to sedation monitoring and the instruments that can be used for monitoring.

### 4.1 Limitations

The findings of this study should be interpreted cautiously due to some limitations. First, the study was only conducted on Finnish wards providing palliative care, which may limit the transferability and international applicability of the findings, as palliative sedation is practised in different ways in different settings. Second, nurses working on the same ward were in the same focus group, which may bias the results, as some nurses spoke extensively whilst others only commented a few times, perhaps because they were a little afraid of expressing their own views. Third, there is no certainty of data saturation, although the same topics clearly began to recur as the interviews progressed. Fourth, the notes taken by the interviewers were not considered in the analysis. Fifth, participants were not asked for feedback on the transcripts or the findings, so it is possible that the themes that were developed do not fully reflect the participant's responses throughout. Finally, the author who analysed the data has previously conducted a literature review on the same research topic, has worked as a nurse on a palliative care ward and has experience in performing palliative sedation, which may, to some extent, affect her interpretation of the data, even if the analysis was discussed in the research group.

## 5 CONCLUSIONS

The findings of this study indicated that nurses play a crucial role in different phases of palliative sedation in palliative care wards in hospitals. The practices of nurses concerning palliative sedation included participation in palliative sedation decision‐making, sedation implementation and monitoring and information sharing and compassionate care for the patient and relatives, in agreement with previous research. Furthermore, this study provided new insights into the contents of these practices, especially on how nurses monitor sedation, what is the role of nurses in emergency sedation, how nurses deliver fundamental care during sedation, what information nurses share with the patient and relatives, and how nurses provide compassionate care to the patient and relatives in the context of palliative sedation.

This study showed that the practices of nurses in palliative sedation should still be clarified, the cooperation between nurses and physicians should be enhanced, and the education provided to nurses on palliative sedation should be increased. Future research should focus on exploring how palliative sedation is monitored, and how the practices of nurses about palliative sedation vary across countries, organizations and cases.

## FUNDING INFORMATION

This study was partly funded by Government research funding (Satakunta Hospital District).

## CONFLICT OF INTEREST

No conflict of interest has been declared by the authors.

## Data Availability

Research data are not shared.
